# The AVRDC – The World Vegetable Center mungbean (*Vigna radiata*) core and mini core collections

**DOI:** 10.1186/s12864-015-1556-7

**Published:** 2015-04-29

**Authors:** Roland Schafleitner, Ramakrishnan Madhavan Nair, Abhishek Rathore, Yen-wei Wang, Chen-yu Lin, Shu-hui Chu, Pin-yun Lin, Jian-Cheng Chang, Andreas W Ebert

**Affiliations:** AVRDC – The World Vegetable Center, P.O. Box 42, Shanhua, Tainan 74199 Taiwan; AVRDC – The World Vegetable Center South Asia, ICRISAT Campus, Patancheru 502 324, Hyderabad, Telangana India; International Crops Research Institute for the Semi-Arid Tropics (ICRISAT), Hyderabad, Telangana India

**Keywords:** Mungbean, Genetic diversity, Germplasm collection, Core and mini core collection, Breeding

## Abstract

**Background:**

Large ex situ germplasm collections generally harbor a wide range of crop diversity. AVRDC – The World Vegetable Center is holding in trust the world’s second largest mungbean (*Vigna radiata*) germplasm collection with more than 6,700 accessions. Screening large collections for traits of interest is laborious and expensive. To enhance the access of breeders to the diversity of the crop, mungbean core and mini core collections have been established.

**Results:**

The core collection of 1,481 entries has been built by random selection of 20% of the accessions after geographical stratification and subsequent cluster analysis of eight phenotypic descriptors in the whole collection. Summary statistics, especially the low differences of means, equal variance of the traits in both the whole and core collection and the visual inspection of quantile-quantile plots comparing the variation of phenotypic traits present in both collections indicated that the core collection well represented the pattern of diversity of the whole collection. The core collection was genotyped with 20 simple sequence repeat markers and a mini core set of 289 accessions was selected, which depicted the allele and genotype diversity of the core collection.

**Conclusions:**

The mungbean core and mini core collections plus their phenotypic and genotypic data are available for distribution to breeders. It is expected that these collections will enhance the access to biodiverse mungbean germplasm for breeding.

**Electronic supplementary material:**

The online version of this article (doi:10.1186/s12864-015-1556-7) contains supplementary material, which is available to authorized users.

## Background

Mungbean, also called green gram [*Vigna radiata* (L.) R. Wilczek var. *radiata*] originated on the Indian subcontinent [1]. From about 1970 onwards, mungbean has been transformed from a marginal, semi-domesticated crop into one of the most important grain legumes in Asia [2]. Mungbean is currently grown on about 6 million hectares, mainly in South and Southeast Asia, but increasingly extends into Australia, USA, Canada and Ethiopia [2]. It is a cheap source of carbohydrates and easily digestible protein [3,4] and contributes folate and iron to the diet, nutrients that often are in short supply in developing countries. Mungbean also fixes nitrogen in the soil, which, together with its short crop duration and low water requirement makes it an important component of crop rotations. Cereals planted after mungbean may yield more and better quality due to the additional nitrogen in the soil [5].

Mungbean landraces have low yields of around 400 kg/ha, while improved varieties can produce more than 2 tons per hectare [6], but viral, bacterial and fungal diseases and insect pests limit commercial yields of mungbean [2]. Current breeding addresses these constraints, targets improved nutritional value, and, due to the expansion of the cultivation range, adaptation of the crop to new environments [2]. The mungbean whole genome sequence became available recently [7], paving the path for molecular breeding approaches that will make trait introgression into elite material more efficient, provided breeders have sufficient access to diverse mungbean germplasm to source these traits.

Ex situ germplasm collections are essential to conserve plant genetic resources for food and agriculture. Mungbean genetic diversity is safeguarded in various germplasm collections; the five largest collections are held at the University of the Philippines; AVRDC – The World Vegetable Center, Taiwan [8]; the Institute of Crop Germplasm Resources of the Chinese Academy of Agricultural Sciences; the All India Coordinated Research Project of the Indian Council of Agricultural Research; and the Plant Genetic Resources Conservation Unit of the University of Georgia, USA [9]. Both the University of the Philippines and the Rural Development Administration (RDA), Korea hold parts of a duplicate of the mungbean germplasm collection of AVRDC – The World Vegetable Center.

Screening of large germplasm collections for traits of interest is laborious and costly. Establishing subsets of collections, so-called core collections, which represent the diversity of the whole collection, makes screening more practical. Mungbean core collections were established in China [10], India [11], the USA [12] and Korea [13]. Molecular analysis of a representative collection of 615 cultivated and wild accessions highlighted the genetic diversity that might be used for broadening the genetic base of mungbean cultivars [14].

Here we describe the establishment and molecular characterization of a core collection derived from AVRDC – The World Vegetable Center’s collection of cultivated mungbean (*Vigna radiata*) and the creation of a mini core collection. The aim of the study is to provide two subsets of germplasm to breeders: a core collection with good representation of the genetic diversity present in the whole collection, and a mini core collection that still maintains a good fraction of the phenotypic diversity of the core collection, and displays a maximum of allele diversity of the larger core.

## Results

### Establishment of a mungbean core collection

A core collection of 1,481 entries was established. The entries and the phenotypic data of the collection are listed in Additional file 1. Comparative analysis of the phenotypic data of the whole and core collection by summary statistics demonstrated that the core collection was highly representative for the whole collection of 5,234 accessions. Especially non-significant *P* values comparing the means and variation of the phenotypic parameters indicated excellent representativeness, although the average Shannon’s index declined from 0.82 in the whole collection to 0.79 in the core (Table 1). The differences of the means between the whole and core collection remained below 1% for all phenotypic descriptors.

**Table 1:** **Means, variances, equality test of two samples (prob > χ2), distance of homogeneity (prob > χ2) and Shannon’s diversity index for the phenotypic values V040, V50, V120, V130, V400, V510, V700 and V770 in the whole collection (WC), the core collection (CC), and the mini core (MC)**

**Variable**	**Means**			**Variances**			**Equality test**			**Distribution of homogeneity**				**Shannon’s diversity index**		
	***WC***	***CC***	***MC***	***WC***	***CC***	***MC***	***WC vs CC***	***WC vs MC***	***CC vs MC***	***Number of classes***	***WC vs CC***	***WC vs MC***	***CC vs MC***	***WC***	***CC***	***MC***
**V040**	4.5	4.5	4.5	0.24	0.23	0.21	0.57	0.71	0.94	11	0.97	0.87	0.76	0.87	0.88	0.83
**V050**	1.7	1.7	1.6	0.05	0.05	0.04	0.97	0.48	0.52	10	0.96	0.54	0.68	0.88	0.87	0.8
**V120**	22.2	22.1	22.6	36.89	38.93	44.45	0.56	0.38	0.27	15	0.8	0.01	0.85	0.79	0.69	0.63
**V130**	40.5	40.6	44.2	149.75	150.39	142.2	0.73	0	0	14	0.88	0	0	0.9	0.89	0.79
**V400**	44.2	44.2	44.9	14.49	15.05	14.06	0.91	0	0	11	0.68	0.06	0.05	0.89	0.86	0.8
**V510**	7.3	7.3	7.3	1.01	1.01	0.76	0.63	0.89	0.92	11	0.95	0.6	0.34	0.74	0.69	0.71
**V700**	11.0	11.0	11.0	1.59	1.64	1.83	0.88	0.28	0.28	13	0.97	0.06	0.12	0.78	0.77	0.75
**V770**	37.5	37.6	36.9	87.24	85.42	87.19	0.61	0.09	0.07	14	0.68	0.22	0.12	0.68	0.7	0.61
**Average**	21.1	21.1	21.6	36.41	36.59	36.34	0.73	0.35	0.38	12.38	0.86	0.3	0.37	0.82	0.79	0.74

**Figure 1:**
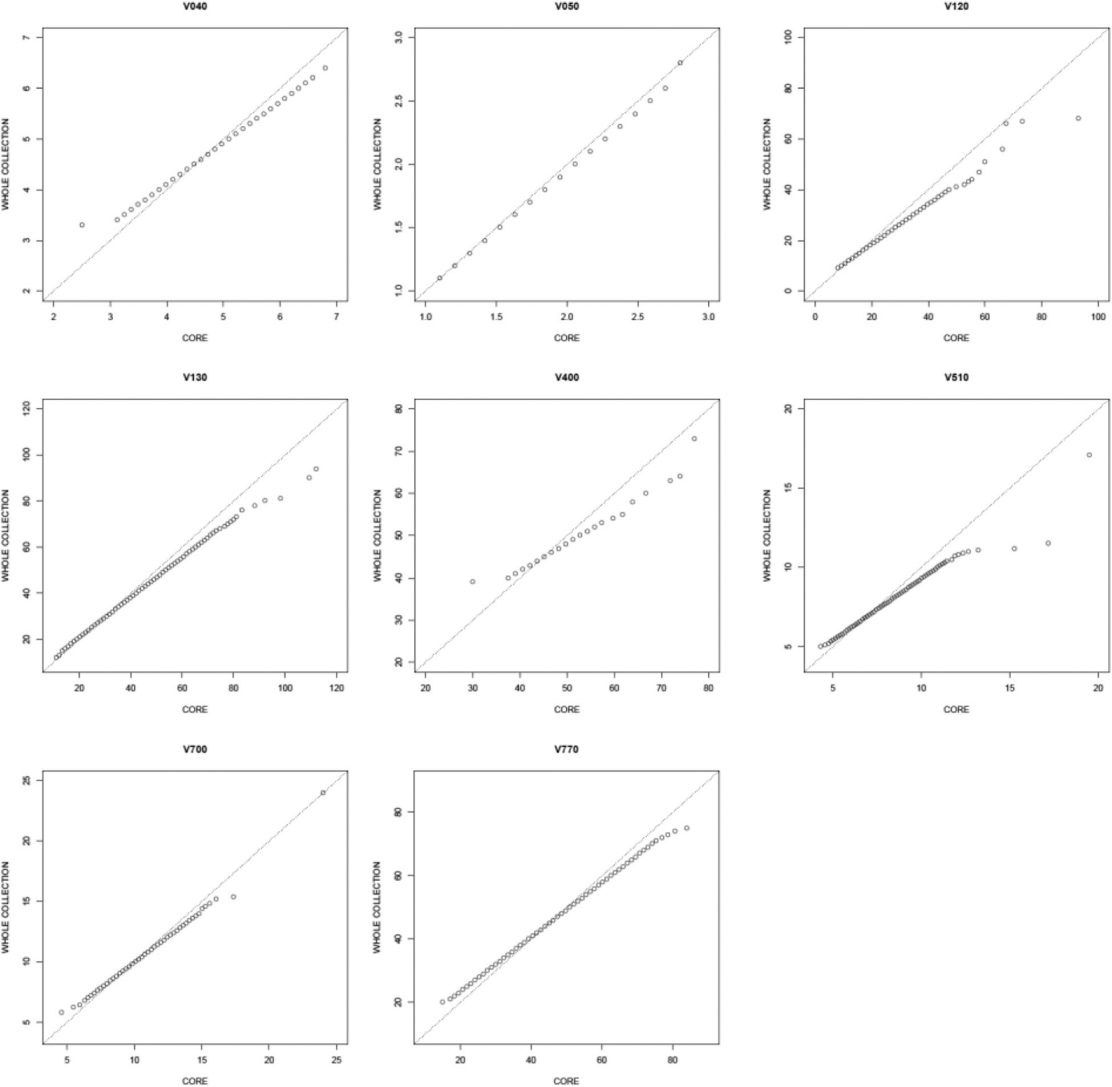
Q-Q plots for the whole and the core collection using phenotypic data sets for V040, V050, V120, V130, V400, V510, V700 and V770.

Visual inspection of quantile-quantile (Q-Q) plots comparing the eight phenotypic data sets confirmed the good representativeness of the core set (Figure 1). Only minor deviations for the upper quantiles of the V120, V130, V400 and V510 data was observed, for the other data sets the value distribution over the quantiles was highly similar between the whole and the core collection.

Unweighted pair-group method using arithmetic means (UPGMA) clustering of the core collection based on phenotypic data is shown in Additional file 2. All except two pairs of entries could be discriminated from each other based on phenotypic values. The dendrogram indicated a clear separation of four core collection entries from the rest of the germplasm accessions at a standardized Euclidean distance of 1.59. These four accessions (602, 1471, 603 and 1490) were characterized by great plant height (V120 and V130), and late flowering (V400). The remaining core set fell into two mega-clusters at a standardized Euclidean distance of 1.18. Around an Euclidian distance of 0.8, one of the mega clusters fell into 6 clusters, and the other into 3. Most distinctions among the germplasm appeared at an Euclidian distance below 0.3.

The evaluation data of the core collection are available through the AVRDC Vegetable Genetic Resources Information System (http://203.64.245.173/). Seed from the collection can be ordered through the AVRDC webpage (http://avrdc.org/seed/seeds/).

### Analysis of the genetic diversity of the core collection based on molecular markers

Out of 400 tested simple sequence repeat (SSR) markers, 20 were chosen to genotype 1,481 accessions of the core collection for their reliable amplification of SSR fragments, for being easy to score, and for having a wide variation of polymorphism information content values when applied on 12 selected mungbean lines (see Additional file 3). The selected SSR markers detected in total 122 different alleles and showed in total 1,387 different genotypes among 1,481 accessions of the core collection. The number of alleles per locus ranged from 3 to 13, with an average of 6.1. (Table 2). The expected heterozygosity (gene diversity, H_T_; [15], defined as the probability that two randomly chosen alleles from the population are different) ranged from 0.145 to 0.707 over all 20 markers (0.485 in average). The Shannon’s information index for each locus was between 0.34 and 1.5 (average 0.851, Table 2). Based on the SSR data, a phylogenetic tree of the core collection was drawn (see Additional file 4).

**Table 2:** **Genotype and allele number per locus in the core (CC) and mini core collection (MC)**

**Locus**	**Observed number of alleles**		**Number of genotypes**		**Shannon’s information index**		**Nei’s expected heterozygosity (Nei’s diversity index)**	
	***CC***	***MC***	***CC***	***MC***	***CC***	***MC***	***CC***	***MC***
AVRDC-MB41	5	5	9	8	1.013	1.108	0.608	0.651
AVRDC-MB44	4	4	7	7	0.913	1.059	0.54	0.636
AVRDC-MB46	6	6	10	10	0.474	0.752	0.266	0.434
AVRDC-MB59	7	7	12	10	0.762	0.856	0.504	0.508
AVRDC-MB60	3	3	4	4	0.681	0.704	0.48	0.502
AVRDC-MB65	4	4	10	10	1.284	1.287	0.707	0.706
AVRDC-MB99	5	5	10	10	0.721	0.837	0.456	0.499
AVRDC-MB148	3	3	4	4	0.694	0.713	0.497	0.503
AVRDC-MB159	5	5	8	8	0.601	0.802	0.354	0.459
AVRDC-MB162	11	10	36	31	1.503	1.74	0.694	0.768
AVRDC-MB180	13	13	24	24	0.796	1.315	0.355	0.585
AVRDC-MB197	8	8	17	16	0.987	1.297	0.496	0.657
AVRDC-MB204	7	7	17	11	1.126	1.185	0.647	0.661
AVRDC-MB241	5	5	8	8	0.342	0.695	0.145	0.34
AVRDC-MB314	8	6	12	10	0.986	1.123	0.582	0.649
AVRDC-MB340	9	7	15	11	0.747	0.92	0.46	0.542
AVRDC-MB347	5	4	9	6	1.209	1.225	0.667	0.68
DMB-SSR80	3	3	5	5	0.595	0.694	0.398	0.472
DMB-SSR125	4	4	8	8	0.691	0.912	0.381	0.503
DMB-SSR130	7	7	14	13	0.904	1.185	0.473	0.604
Mean	6.1	5.8	12	10.7	0.851	1.02	0.485	0.568
STDEV	2.7	2.5	7.5	6.5	0.284	0.279	0.145	0.108

A few core collection accessions were highly distinct from the others, as shown by the dendrograms of phenotypic and genotypic diversity. The most distant group consisted of the three entries no. 1470, 685 and 623, representing a local cultivar from Taiwan (VI005024) and the varieties JMP 1972 (VI002529) from Thailand and 372-M (VI002274) from Afghanistan. Most of the remaining accessions fell into two mega clusters, similar to the dendrogram for phenotypic data. Only ten accessions formed three additional side groups to the two main mega clusters (see Additional file 4).

We tried to measure the correlation between the phenotypic and genotypic dendrograms. For this purpose, a Mantel test comparing the diversity matrices of the phenotypic and genotypic data was performed. The correlation detected was very small (*R*_*xy*_ = 0.149), but significant (*P* = 0.001), while correlation analysis in MXCOMP resulted in a significant negative correlation between the matrices. Visual inspection of the dendrograms showed that while the grouping of the accessions to the mega clusters was consistent between the dendrograms for phenotypic and genotypic similarity, the arrangement of the entries in the subgroups was highly different.

**Figure 2:**
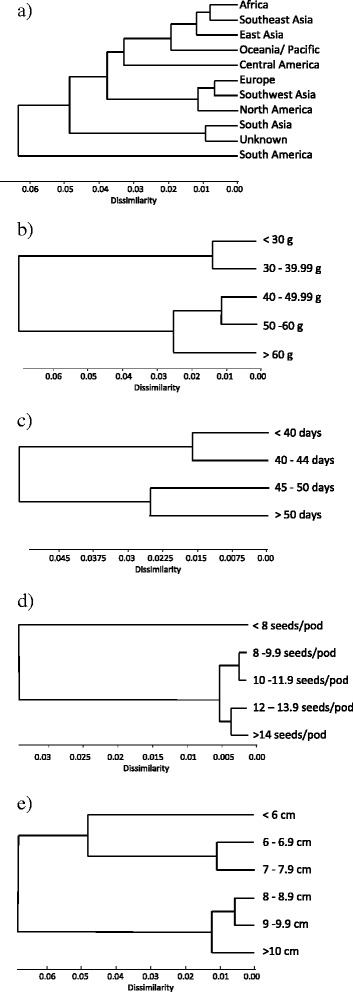
Dissimilarities of molecular marker genotypes for core collection subgroups formed by **a)** geographic origin, **b)** 1000-seed weight (V770, <30 to 60 g), **c)** flowering time (V400, <40 to > 50 days), **d)** seed number per pot (V700, <8 to >14 seed per pod) and **e)** pod length (V510, <6 to >10 cm).

Correlations between geographical origin or phenotypic characteristics and the genetic similarity in terms of molecular marker genotype were investigated. South American accessions were genetically more distant from any other geographical group, while accessions derived from Europe, Southwest Asia and North America appeared to be more related (Figure 2a). The genetic distance found between accessions grouped by 1000 seed weight, time to flowering and pod length indicated some congruence between phenotypic values and marker genotype (Figure 2b, c and e). For example, SSR genotyping separated accessions with smaller seeds (less than 54 g per 100 seeds) from larger-seeded entries (Figure 2b). The same was true for early and late flowering accessions and small and large-podded plants, where groups with contrasting phenotype were also separated by genotype (Figure 2c). For seed number per pod, only those accessions with less than 8 seeds per pod were separated from those with 8 to more than 14 seeds based on SSR marker genotype (Figure 2d).

Additionally, the population structure of the core collection was investigated for K values from 1 to 50. Similar to the UPGMA analyses for phenotypic and genotypic similarity, the uppermost level of structure of the core collection fell into 2 distinct sub-populations. Subsampling individuals of largest and smallest 1000 seed weight (upper-most and lowest branch of the dendrogram of Figure 2b also led to K = 2, suggesting 2 different sub-populations.

### Establishment and analysis of a mini core collection

Based on the genotypic data of the core collection, a mini core collection was drawn that contained 20% of the core collection and about 4% of the total collection. The accessions of the mini core are listed in Additional file 1. It showed that all geographic regions represented in the core set were also present in the mini core set, although in slightly different proportions. As for the core collection, summary statistics for the phenotypic descriptors V040, V050, V120, V130, V400, V510, V700 and V770 (Table 1), as well as for genotypic parameters (Table 2) were analyzed. The differences between the mean values for the phenotypic descriptors of the mini core and core set increased compared to the difference between the core and whole collection, but remained under 1% for four of the eight descriptors, and reached a maximum of 9% for V130 (plant height at maturity). Variances of the phenotypic descriptors increased up to 24% when compared to the core set, as shown by significant values for the distribution of homogeneity for comparisons with the whole collection (V120, V130) and the core collection (VV130, V400), and significant values for the equality test for V130 and V400 (Table 1). The average Shannon’s index of the phenotypic diversity declined from 0.79 in the core collection to 0.74 in the mini core, indicating some loss of diversity through reduction of the number of entries. On the molecular level, the mini core contained in total 6 alleles less than the core and the average allele number was reduced from 6.1 to 5.8 (Table 2). The 296 entries had 294 different SSR marker genotypes, only one pair of accessions shared the same marker genotype. As expected for a smaller set designed to represent a larger one, the average Nei’s and Shannon’s diversity indices increased in comparison to the core set. Genotypic and structure analysis using SSR data suggested the presence of two major subgroups in the mini core, while diversity analysis using phenotypic data indicated the presence of three subgroups (see Additional files 5 and 6).

**Figure 3:**
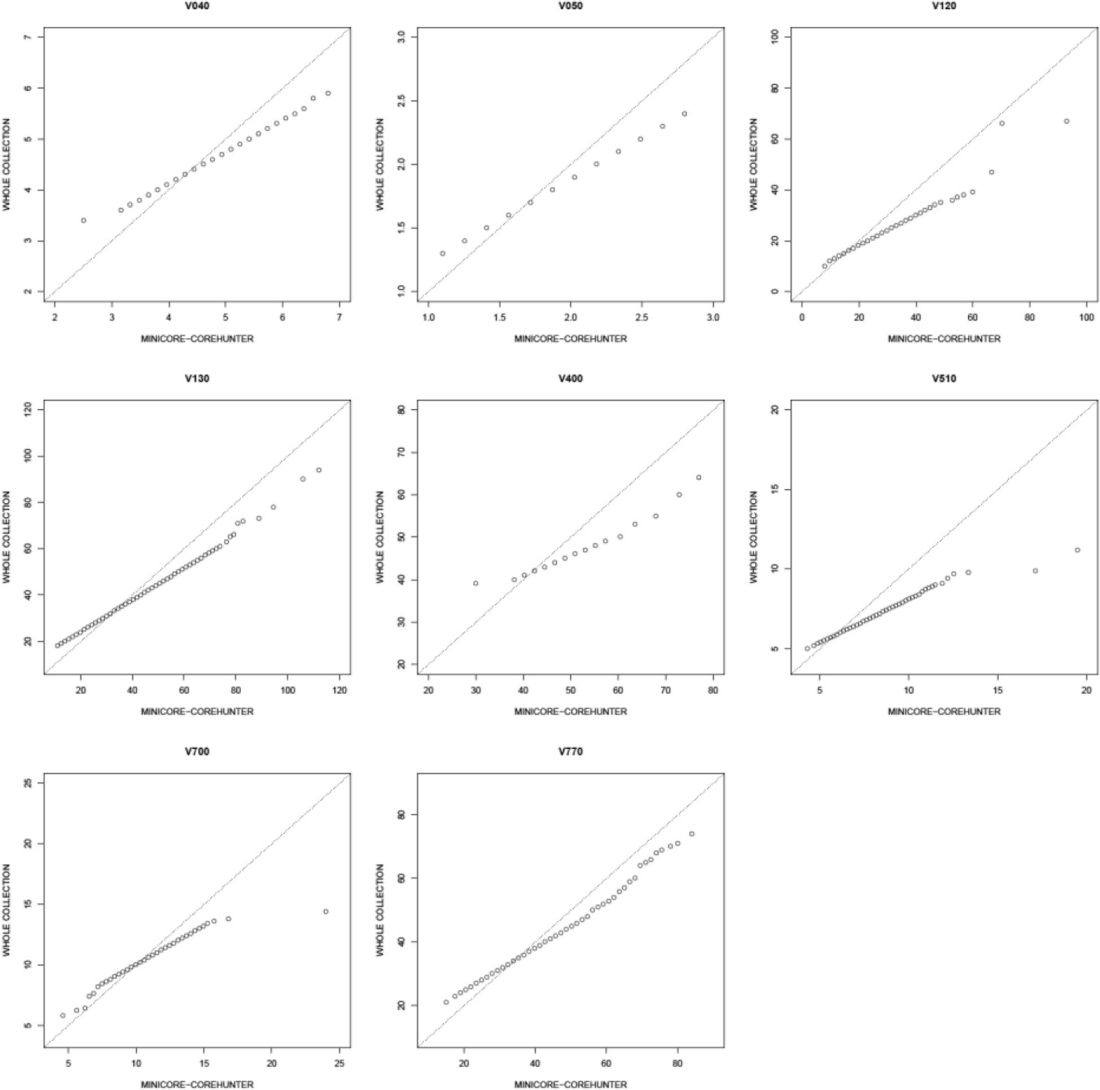
Q-Q plots for the whole and the Core Hunter mini core collection using phenotypic data sets for V040, V050, V120, V130, V400, V510, V700 and V770.

Q-Q plots suggested greater deviation of the value distribution for the phenotypic datasets between the whole and mini core collection than observed in the comparison of the core collection with the whole (Figure 3).

To validate the method applied to extract the mini core from the core collection, two more mini core sets of the same size were established, one by random drawing of 20% of the accessions from each cluster obtained by SSR diversity analysis of the core collection, and one by stratification of the core collection based on geographical origin and random selection of accessions from each geographical cluster. Neither alternative mini core set had better summary statistics scores for phenotypic parameters than the mini core constructed by Core Hunter software. The significance test of two population correlation coefficients between the core collection and the Core Hunter mini core was significant at p = 0.05 for one descriptor (V700), while it was significant for four descriptors for the alternative mini cores. On the molecular level, the best alternative mini core had in average 0.6 alleles and 1.7 genotypes per marker less than the mini core established by Core Hunter (Table 3).

**Table 3:** **Allele and genotype numbers in the Core Hunter mini set (MC core hunter) compared with a mini core based on regional stratification (MC-region) or by random selection from the core collection (MC-random)**

**Locus**	**Observed number of alleles**				**Number of genotypes**			
	**Core**	**MC Core Hunter**	**MC-region**	**MC-random**	**Core**	**MC Core Hunter**	**MC-region**	**MC-random**
AVRDC-MB41	5	5	3	4	9	8	6	7
AVRDC-MB44	4	4	3	3	7	7	6	6
AVRDC-MB46	6	6	4	6	10	10	5	7
AVRDC-MB59	7	7	6	6	12	10	7	7
AVRDC-MB60	3	3	3	3	4	4	4	4
AVRDC-MB65	4	4	4	4	10	10	10	10
AVRDC-MB99	5	5	4	5	10	10	5	8
AVRDC-MB148	3	3	2	2	4	4	3	3
AVRDC-MB159	5	5	4	5	8	8	7	6
AVRDC-MB162	11	10	8	9	36	31	23	28
AVRDC-MB180	13	13	7	12	24	24	14	19
AVRDC-MB197	8	8	7	6	17	16	14	12
AVRDC-MB204	7	7	4	6	17	11	8	9
AVRDC-MB241	5	5	4	5	8	8	6	7
AVRDC-MB314	8	6	6	4	12	10	9	7
AVRDC-MB340	9	7	5	6	15	11	8	9
AVRDC-MB347	5	4	4	4	9	6	6	6
DMB-SSR80	3	3	2	3	5	5	3	5
DMB-SSR125	4	4	4	4	8	8	7	7
DMB-SSR130	7	7	5	7	14	13	9	13
*Mean*	*6.1*	*5.8*	*4.5*	*5.2*	*12.0*	*10.7*	*8.0*	*9.0*

## Discussion

According to [16], germplasm collections must serve both the current requirements of breeders, as well as preserve genetic resources for future needs. Germplasm characterization is essential to identify the right material for breeders and for making the correct decisions for germplasm conservation and collection activities. Screening large collections for traits of interest is economically and logistically challenging, and is generally too laborious and costly for most breeding programs. Consequently, crop diversity remains locked in genebanks, while breeders depend on sourcing germplasm from small working collections, resulting in a narrow genetic base for the crop and lack of genes for disease resistance and quality traits. Mainly due to this constraint, mungbean breeding in the past has relied on a small number of lines, resulting in varieties that are genetically related and lack resistances to pests, diseases and abiotic stresses.

Core collections facilitate access to genetically diverse germplasm and trait diversity. They comprise a manageable number of accessions, while retaining the greatest part of the genetic variability found in large germplasm collections [17,18]. Their reduced size of 5 to 20% of the entire collection facilitates germplasm screening and evaluation, leading to a better understanding of the genetic structure of the crop species and promoting the distribution of information and plant material for breeding. For large collections, establishing mini cores makes germplasm screening even more workable.

Approaches to generate core collections generally apply a combination of geographical, morphological, agronomic and molecular characteristics for stratification of genebank collections to select entries that are considered to represent the diversity of the whole collection. Core collections established through a combination of genotypic data (e.g. SSR markers) and phenotypic data, are thought to contain larger genetic variability and have superior representativeness than those based on phenotypic values alone [19,20]. Molecular markers are highly suitable tools to assess the diversity within groups or the divergence between them, but their application might be prohibitively laborious and expensive for very large collections of minor crop species. Genotyping of the whole mungbean collection currently consisting of more than 6,700 accessions was out of scope for the present study, therefore the AVRDC – The World Vegetable Center mungbean core collection was established by geographical stratification of the whole collection comprising more than 5,000 accessions and subsequent diversity analysis based on eight phenotypic descriptors. Only at the level of the core collection molecular characterization became feasible. However, neither mapped SSR markers nor the full genome of mungbean was available at the time the genotyping effort was accomplished, thus it was not possible to select markers that were evenly distributed over the mungbean genome. Nevertheless, the selected 20 markers, according to recent mapping of these SSRs to the mungbean genome, were located on 7 of the 11 chromosomes of mungbean and additionally cover three sequence scaffolds that have not yet been mapped to any of the chromosomes [7]. Therefore it is assumed that most of the 20 markers are unlinked and thus have been suitable to assess the genetic diversity of the core set.

Evaluation of the quality of the core and mini core collection required the choice of the right criteria for the kind of core collection to be analyzed [21]. The core collection of the present study should represent the pattern of variation present in the whole collection, while the mini core should conserve a maximum of the variation present in the core collection, but also should capture the rare or extreme traits, e.g. high resistance to insect pests and diseases or high yield. The quality assessment of the core collection should test whether the distribution of the phenotypic traits is similar to that of the whole collection. Summary statistics and Q-Q tests corroborated the similar distribution of trait variation in both the whole and core collection. Means of the phenotypic parameters assessed in both the whole and core collection essentially remained unchanged after reduction of the number of entries from more than 7,000 to 1,481, and the largest change of variance between the collections was 5.5% for V120. Equality and homogeneity test remained insignificant for all parameters, and the decrease of the Shannon’s diversity index for the phenotypic values was very modest when compared to the whole collection. However, it should be noted that the Shannon’s diversity index is rejected by some authors as an indicator of diversity, as it is sensitive to small fractions and has no direct meaning [22]. Q-Q plot analysis also suggested that the core collection represented the trait diversity of the whole collection. Diversity analysis on both the phenotypic and the molecular level suggested that the core collection is distributed over two mega clusters. The split of the core into two distinct populations was confirmed by population structure analysis and was also largely conserved in the mini core collection, which was extracted from the core collection based on genotypic data.

Analysis of the mini core collection focused on the conservation of allele richness and genotype diversity compared to the core collection, but the conservation of trait variability of the whole and core collection in the mini core was also tested. Summary statistics in the mini core, specifically the equality test and distribution of homogeneity test suggested significant differences for the distribution of two out of the eight phenotypic descriptors between the core and whole population. The Shannon’s diversity index for the phenotypic descriptors decreased in the mini core, but the average drop from the core to the mini core collection was very small. Algorithms for selection of core collections such as Core Hunter are designed to maximize genetic diversity parameters such as allelic richness and thus are likely to select also non-representative “outlier” accessions [21]. Therefore it was expected that the allele richness and genotype diversity between the core and mini core collections was very similar. Comparison of random selection of accessions from genetic distance clusters or from the core collection stratified by geographical origin revealed that they were inferior to the mini core produced by Core Hunter. Both number of alleles and number of genotypes was decreased in the alternative mini core sets compared to the Core Hunter mini core.

The correlation between the dendrograms of phenotypic and genotypic similarity of the core set was very low (near 0 by Mantel test), or even negative, when the MXCOMP was used, but in both cases the *P*-value was highly significant, so we could not draw any conclusion about congruence of phenotypic with genotypic diversity. But grouping of core collection accessions based on phenotypic characteristics including seed weight, pod size and seeds per pod showed differences in SSR genotype between the phenotypic groups, indicating some degree of consistency between phenotypic and genotypic diversity in the core set. In the mini core this kind of test has not been performed due to the relatively small number of accessions per phenotypic subgroup.

## Conclusions

The core and mini core collection, together with the evaluation and genotypic data, is now available for distribution to breeders. Continuous evaluation of the core collection for traits of interest for breeding such as biotic and abiotic stress tolerance will add information and thus value to these collections. Based on the genotypic and phenotypic data available for the collections, more mini cores can be produced, either by applying alternative methods to select entries, such as PowerCore [23] or mini cores enriched for specific traits that may serve specific environments.

## Methods

### Establishment of the core collection

The whole mungbean collection as available in 1984 consisted of 5,234 accessions, or 7,965 entries when variants sorted by seed luster and color were included, was stratified based on the geographical origin of the accessions. The current collection consists of 6,742 accessions or 9,649 entries, when sub-accessions are included. The countries of origin of the accessions were grouped into regions, and then the accessions of each region were clustered based on eight phenotypic descriptors measured at AVRDC – The World Vegetable Center during the years 1984 (spring and fall seasons) and 1985 (fall season): primary leaf length in cm (V040), primary leaf width in cm (V050), plant height at flowering in cm (V120), plant height at maturity in cm (V130), days to 50% flowering (V400), pod length in cm (V510), seeds per pod (V700) and 1000 seed weight in g (V770). The data were standardized and submitted to similarity analysis using the Euclidian coefficient and clustering according to [24] in NTSYS-Spec 2.11 L. From each cluster, 20% of the accessions were randomly selected to constitute a core set of 1,481 entries.

### Selection of molecular markers and genotyping

Genomic DNA was isolated from fresh leaf tissue using the protocol of [25]. SSR primer sequences were obtained from [26] and [27]. Additionally, shot-gun DNA sequences from [28] were assembled using CAP [29] and mined for microsatellite motifs by the SSR-Locator software [30]. In total, 400 markers with di- and tri-mer repeats were randomly chosen for wet-lab testing on 11 mungbean breeding lines (VGG80, VGG04.025, SU4-146, VGG 04. 023, AVMU9701, AVMU0001, AVMU0002, AVMU0201, AVMU0401, AVMU8601, VC6141-54) and one genebank accession (VI059227). Twenty SSR markers that amplified reliably across these lines, were easy to score and had a large variation in polymorphism information content value as determined in PowerMarker according to [31], were selected to amplify SSRs from the accessions of the core collection (see Additional file 1). Once the mungbean whole genome sequence became available [7], the sequences of the SSR primers were mapped to the genome sequence using blast.2.2.27+ [32] to determine the most probable chromosomal location of the markers.

Polymerase chain reaction (PCR) amplifications were performed in 15 μl reactions containing 0.2 μM of each primer, 200 μM of deoxyribonucleotides, 50 mM KCl, 10 mM Tris HCl (pH 8.3), 1.5 mM MgCl_2_, 25 ng of DNA and 0.5 unit of *Taq* super-therm gold DNA polymerase JMR-851 (Bertec, Taiwan). The SSR amplifications were conducted in PTC 200 DNA engine thermal cyclers (MJ Research, USA). The temperature profile used for PCR amplification was 94°C for 5 min, followed by 30 cycles of 94°C for 30 s, 55°C for 45 s, 72°C for 45 s, and finally by 7 min at 72°C for the final extension. Annealing temperature was adjusted based on the specific requirement of each primer combination. PCR products (3 μl) were analyzed on 6% non-denaturing polyacrylamide gels in 0.5× Tris-borate-EDTA buffer. After electrophoresis, the gels were stained with 5 μg/ml^−1^ ethidium bromide and the bands were visualized under ultraviolet light using the alpha imager system. Presence and absence of DNA fragments were scored as 1 and 0, respectively. The number of alleles, genotype diversity, Shannon’s and Nei’s diversity indices were determined in PopGene 1.32 [33] for each marker.

### Phenotypic and genotypic characterization of the core collection

The phenotypic values for the descriptors V040, V050, V120, V130, V400, V510, V700 and V770 of the core collection were processed by the NTSYS-Spec 2.11 L software. First the data were standardized to account for the different scales of measurement. A similarity matrix was generated using the Euclidian coefficient, cluster analysis was performed by the group average method (UPGMA-unweighted pair-group method using arithmetic means) and a dendrogram was generated to depict the inter-relationships among the accessions of the core collection. The genotyping data of the core collection were used for a pairwise genetic similarity determination among entries of the core collection using the Jaccard coefficient in the SIMQUAL module of NTSYS-Spec 2.11 L. Phylogenetic trees were constructed using UPGMA of the SAHN module of the software. The relationship between the phenotypic and genotypic diversity of the core collection was assessed by a paired Mantel test [34] using GenAlEx 6.5 [35] with 999 permutations and by the MXCOMP module of NTSYS-Spec 2.11 L computing the correlation between the phenotypic and genotypic diversity matrices. Nei’s original measures of genetic identity and genetic distance [15] called Nei’s diversity index was calculated for the SSR data in PopGene 1.32 [33]. The population structure of the core and mini core collection was analyzed in the program Structure 2.3.4 [36] using a burn-in period and MCMC repeats after burn-in of 50,000 using an admixture model. The data obtained by Structure 2.3.4 were analyzed for optimal K values by the Structure Harvester [37].

### Extraction of mini core collection

The Core Hunter software [38] was run on Ubuntu 12.04 on genotypic data of 1,481 entries of the core collection using the default Mixed Replica Algorithm optimizing the Modified Rogers’ distance (weight 0.7) and Shannon’s diversity index (weight 0.3) to define a mini core comprising about 20% of the entries of the core collection. The genotypic diversity was analyzed in PopGene 1.32, and establishment of dendrograms was performed as described for the core collection.

### Comparisons of the whole collection with the core and mini core collections

Summary statistics were applied to evaluate the representativeness of the core and mini core collections: Means, Variance, Equality (χ^2^ test), Karl Pearson’s correlations and Shannon’s diversity index were calculated for the whole collection, the core and the mini core set in SAS (SAS Institute Inc., Cary, NC, USA). Q-Q plots were constructed comparing the distribution of eight phenotypic data sets (V040, V050, V120, V130, V400, V510, V700 and V770) of the whole collection, the core and the mini core set over 0.5% quantiles in R (http://www.r-project.org/) and the Kullback–Leibler distance between the values generated by the Q-Q plot were calculated using the KL.Plugin function of the EntropyEstimation package in R [39].

### Availability of supporting data

All supporting data are included as additional files. The passport and evaluation data of the accessions of the core and mini core sets are available in the AVRDC Vegetable Genetic Resources Information System at http://203.64.245.173/.

## Additional files

Additional file 1:
**List of entries and phenotypic data of the AVRDC - The World Vegetable Center mungbean core and mini core collections.** Entries of the mini core are labeled with MC in the Mini core column. Origin: AFR: Africa; EUR: Europe; MA: Central America; NA: North America; OP: Ozeania and the Pacific; SA: South Asia; SAM: South America; SEA: South East Asia; SWA: South West Asia; UK: unknown. The descriptors V040 to V770 are explained in Methods. Additional file 2:
**Dendrogram depicting the relatedness among the entries of the core collection based on phenotypic data.**
Additional file 3:
**SSR primers, PIC and genomic location used for genotyping of the mungbean core collection.**
Additional file 4:
**Dendrogram of the core collection entries based on their SSR genotype.**
Additional file 5:
**Dendrogram of the mini core collection based on the diversity of the phenotypic values for V040, V050, V120, V130, V400, V510, V700 and V770.**
Additional file 6:
**Dendrogram of the mini core collection based on SSR marker analysis.**

## Abbreviations

CC: Core collection
MC: Mini core collection
PCR: Polymerase chain reaction
Q-Q plots: Quantile-quantile plots
SSR markers: Simple sequence repeat markers
UPGMA: Unweighted pair-group method using arithmetic means
WC: Whole collection
