# Supplementary material for: The AVRDC – The World Vegetable Center mungbean (Vigna radiata) core and mini core collections
Source: BMC Genomics. 2015 Apr 29;16(1):344. doi: 10.1186/s12864-015-1556-7 (PMC4422537; doi:10.1186/s12864-015-1556-7)
Supplement: Additional file 2: — Dendrogram depicting the relatedness among the entries of the core collection based on phenotypic data. [file 12864_2015_1556_MOESM2_ESM.docx]

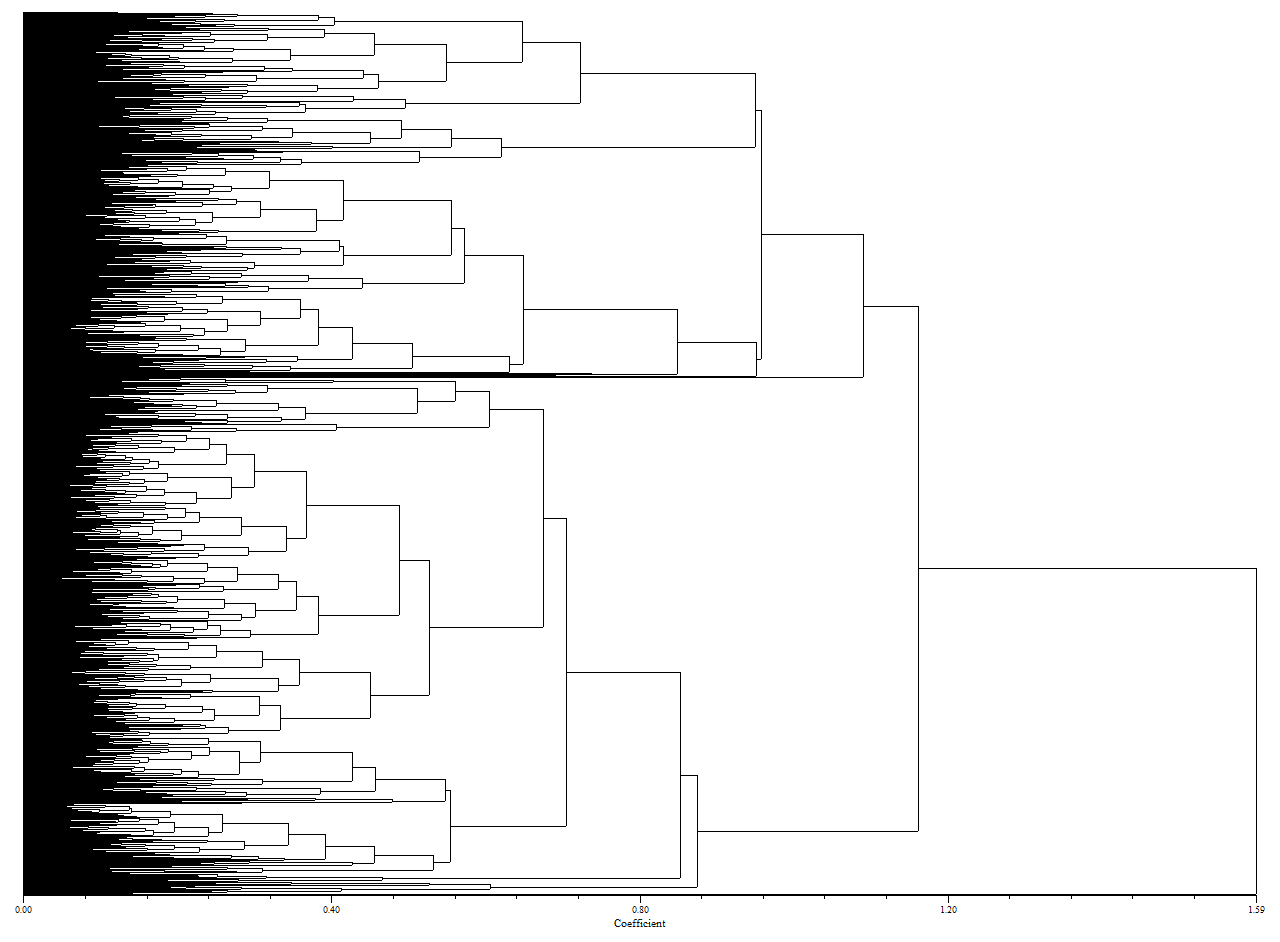


Additional file 2: Dendrogram depicting the relatedness among the entries of the core collection based on phenotypic data.
