# Supplementary material for: The AVRDC – The World Vegetable Center mungbean (Vigna radiata) core and mini core collections
Source: BMC Genomics. 2015 Apr 29;16(1):344. doi: 10.1186/s12864-015-1556-7 (PMC4422537; doi:10.1186/s12864-015-1556-7)
Supplement: Additional file 3: — SSR primers, PIC and genomic location used for genotyping of the mungbean core collection. [file 12864_2015_1556_MOESM3_ESM.docx]

Additional file 3: SSR primers, PIC and genomic location used for genotyping of the mungbean core collection.

| **Marker** | **PCR Primer** | **PIC** | **Reference** | **Position** |
| --- | --- | --- | --- | --- |
| AVRDC-MB41 | Fw: TGAGCCCACGTTGAAGTTAG  Rev: CCATTGACGACTCTTGCAGT | 0.53 | AVRDC, unpublished from sequences published by Moe et al., 2011 | Chr. 06 |
| AVRDC-MB44 | Fw: ATTGCTCCAGCAGGGTTTAT  Rev: AAGCAACACTATGCAGCAGG | 0.47 |  | Chr. 08 |
| AVRDC-MB46 | Fw: ACTCACCAATTTCCCAGAGG  Rev: CGTTCAGCATCAATTCCATC | 0.23 |  | Scaffold 303 |
| AVRDC-MB59 | Fw: AGAAGGCTCTCAAGCAGAGG  Rev; CAATAACCAGCCGTCAGCTA | 0.39 |  | Chr. 08 |
| AVRDC-MB60 | Fw: GCACTTGATGCCACGTAGTT  Rev: ACTTGAAGTGCCCTTGTTTG | 0.37 |  | Chr. 01 |
| AVRDC-MB65 | Fw: CCTCAGCAAGAAAGGAAAGG  Rev: ACCCTCTGAGTTCATCGTCC | 0.65 |  | Chr. 02/ scaffold 246 |
| AVRDC-MB99 | Fw: GTCGAAGCCCAGAAAGAGTC  Rev: TTTCGATCAGGAAGCTGTTG | 0.37 |  | Chr. 10 |
| AVRDC-MB148 | Fw: GGTTTGGGAATGTGTGTGAG  Rev: CAACAAGGACAAGGACATGG | 0.37 |  | Chr. 05 |
| AVRDC-MB159 | Fw: CCTATGTGATGAGGCTTCCA  Rev: ATTGAGAGAGGAGGCAGTGG | 0.3 |  | Chr. 07 |
| AVRDC-MB162 | Fw: CAAGGCACTTCCTTAAACCC  Rev: AGGACCCTACTGTTTGTGTTGA | 0.66 |  | Scaffold 7 |
| AVRDC-MB180 | Fw: AAGCATAATTAACTGGCGGG Rev: GTCTTGGCAGGTTCCATCTT | 0.34 |  | Chr. 02 |
| AVRDC-MB197 | Fw: TCTCTCTCAGTCCAAATCACAC  Rev: CGAGGCTTTTCCATGAAACT | 0.46 |  | Chr. 05 |
| AVRDC-MB204 | Fw: ACGCCAAACTAAAACCGAGT  Rev: GCCGATCCCATTTTCAAG | 0.56 |  | Scaffold 73 |
| AVRDC-MB241 | Fw: TTCTGAACCTGTCTGTCCCA  Rev: CAATCCACACACACTTCAAGG | 0.14 |  | Chr. 05 |
| AVRDC-MB314 | Fw: GAGGAAAACCTGCAACAACA  Rev: ATTCTAGGTCCGTTCCTCCC | 0.50 |  | Chr. 04 |
| AVRDC-MB340 | Fw: TTCCTTAGCTTCAAGGGCAC Rev: TGGTTGTCAGGCCTTATTTG | 0.37 |  | Chr. 05 |
| AVRDC-MB347 | Fw: TGCACCTAAATCTTGCAACC  Rev: GTCCATTGCCATTGTTGTGT | 0.56 |  | Chr. 07 |
| DMB-SSR80 | Fw: CGAGGCAGAGAAACCTTAAGAA Rev: GCTCGATACTCTTGGGTTGAA | 0.32 | Somta et al., 2009 | Chr. 10 |
| DMB-SSR125 | Fw: AAAATGAGTGACAGAGGTGGAAA  Rev: ACATGCACATTCTGAACCACAT | 0.34 |  | Chr. 04 |
| DMB-SSR130 | Fw: CAACTGCAAATGAGGTGAAGAT  Rev: ATCCAAGAGCATTTGAACTTCC | 0.43 |  | Chr. 09 |
