# Supplementary figures and images for: The AVRDC – The World Vegetable Center mungbean (Vigna radiata) core and mini core collections
Source: BMC Genomics. 2015 Apr 29;16(1):344. doi: 10.1186/s12864-015-1556-7 (PMC4422537; doi:10.1186/s12864-015-1556-7)

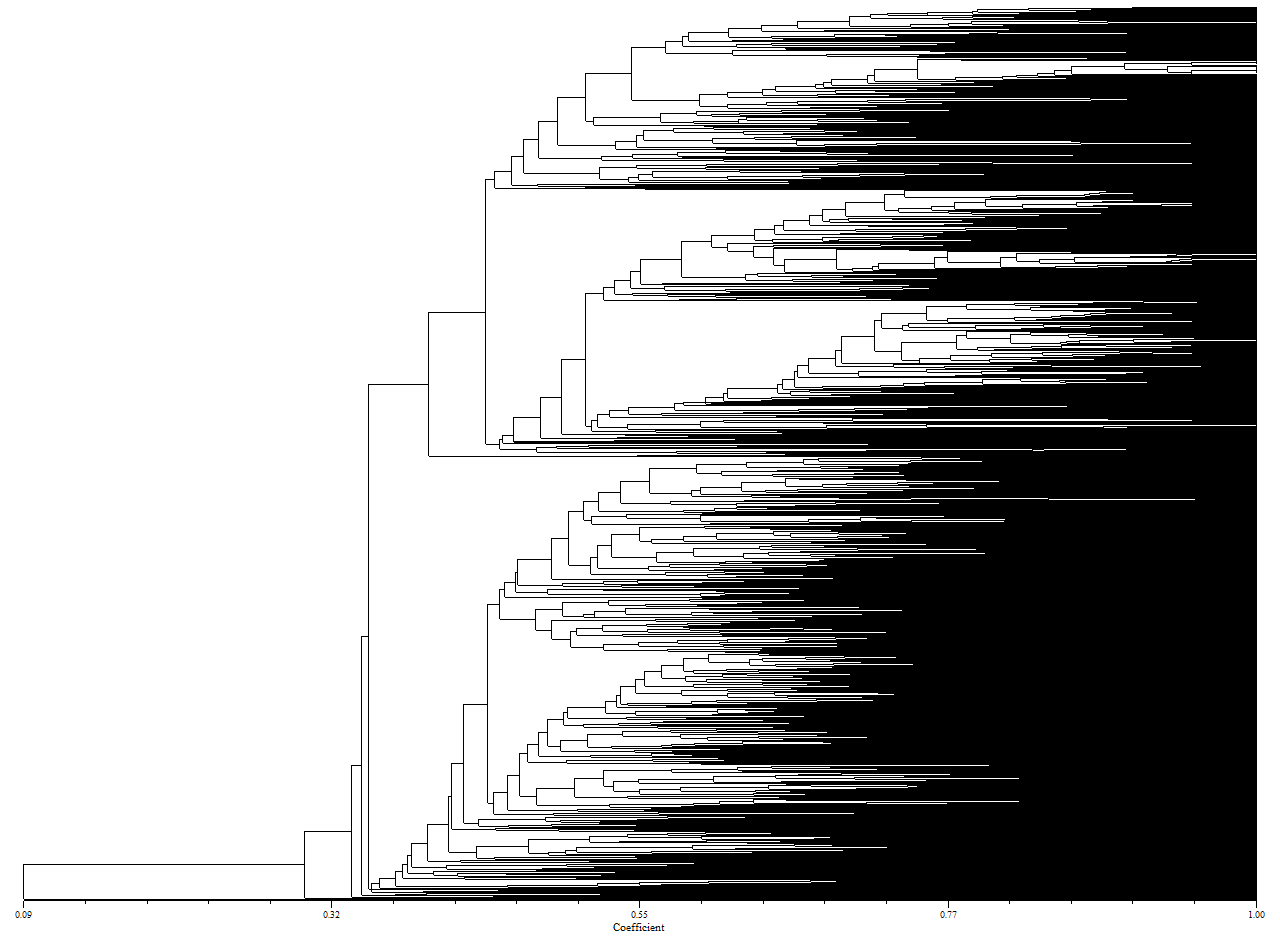


Additional file 4: Dendrogram of the core collection entries based on their SSR genotype.

Supplement: Additional file 4: — Dendrogram of the core collection entries based on their SSR genotype. [file 12864_2015_1556_MOESM4_ESM.docx]

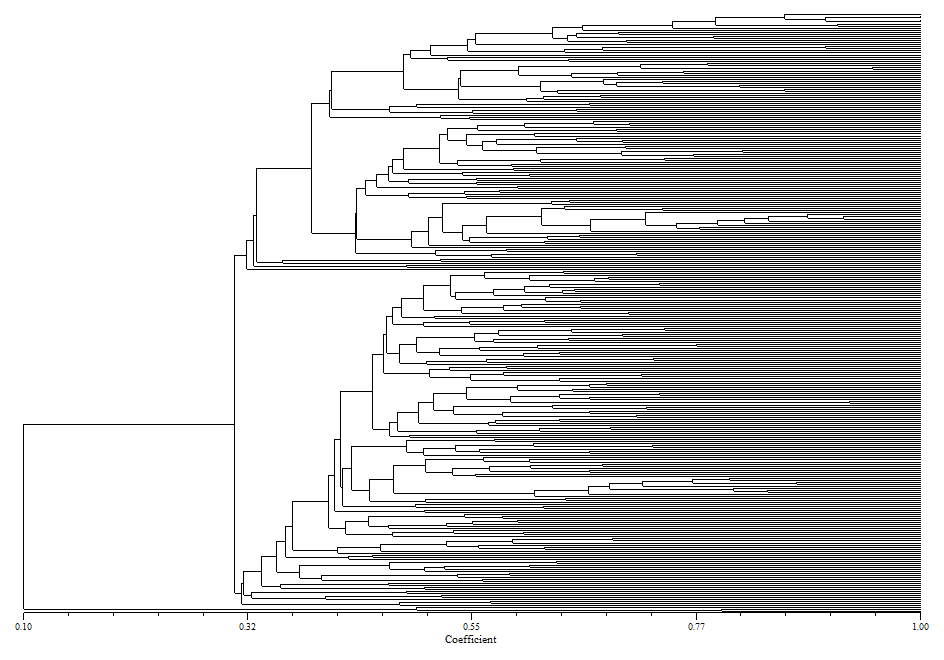


Additional file 6: Dendrogram of the minicore collection based on SSR marker analysis.

Supplement: Additional file 6: — Dendrogram of the mini core collection based on SSR marker analysis. [file 12864_2015_1556_MOESM6_ESM.docx]
