# Supplementary material for: The AVRDC – The World Vegetable Center mungbean (Vigna radiata) core and mini core collections
Source: BMC Genomics. 2015 Apr 29;16(1):344. doi: 10.1186/s12864-015-1556-7 (PMC4422537; doi:10.1186/s12864-015-1556-7)
Supplement: Additional file 5: — Dendrogram of the mini core collection based on the diversity of the phenotypic values for V040, V050, V120, V130, V400, V510, V700 and V770. [file 12864_2015_1556_MOESM5_ESM.docx]

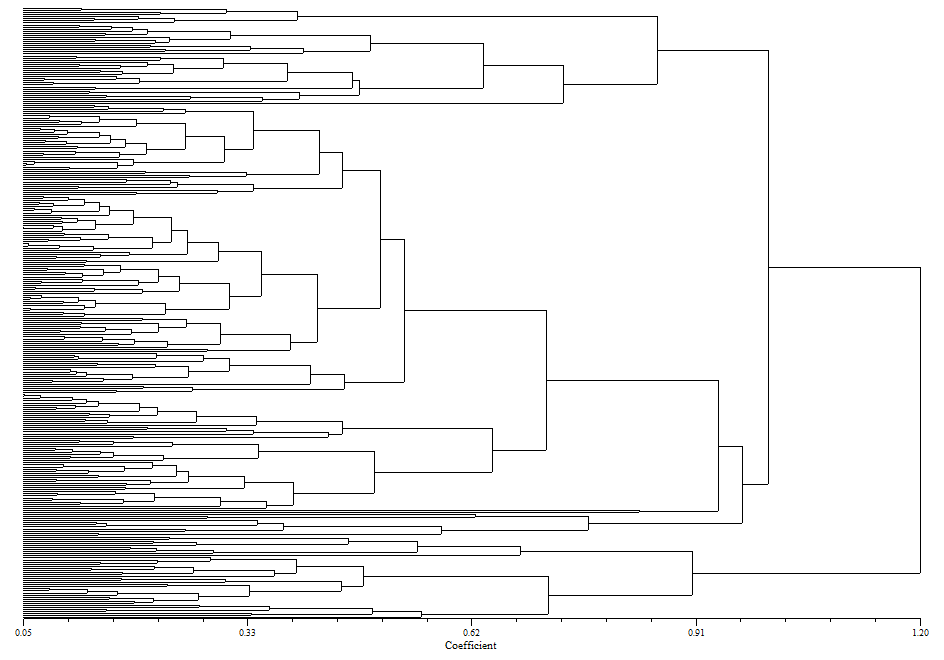


Additional file 5: Dendrogram of the minicore collection based on the diversity of the phenotypic values for V040, V050, V120, V130, V400, V510, V700 and V770.
